# Asymptomatic bacteriuria in type 2 Iranian diabetic women: a cross sectional study

**DOI:** 10.1186/1472-6874-6-4

**Published:** 2006-02-23

**Authors:** Mohammad Ali Boroumand, Leila Sam, Seyed Hesameddin Abbasi, Mojtaba Salarifar, Ebrahim Kassaian, Saeedeh Forghani

**Affiliations:** 1 Tehran Heart Center, Tehran University of Medical Sciences, Tehran, Iran; 2 Social Security Organization, Karaj, Iran; 3 National Iranian Oil Company Central Hospital, Tehran, Iran; 4 Razi Institute, Karaj, Iran

## Abstract

**Background:**

The risk of developing infection in diabetic patients is higher and urinary tract is the most common site for infection. Serious complications of urinary infection occur more commonly in diabetic patients. To study the prevalence and associates of asymptomatic bacteriuria (ASB) in women with type 2 diabetes mellitus in the Iranian population, this study was conducted.

**Methods:**

Between February 10, 2004 and October 15, 2004; 202 nonpregnant diabetic (type 2) women (range: 31 to 78 years old) with no abnormalities of the urinary tract system were included in this clinic based study. We defined ASB as the presence of at least 10^5 ^colony-forming units/ml of 1 or 2 bacterial species, in two separated cultures of clean-voided midstream urine. All the participants were free from any symptoms of urinary tract infection (UTI). Associates for developing bacteriuria was assessed and compared in participants with and without bacteriuria.

**Results:**

In this study, the prevalence of ASB was 10.9% among diabetic women. E. coli was the most prevalent microorganism responsible for positive urine culture. Most of the isolated microorganisms were resistant to Co-trimoxazole, Nalidixic acid and Ciprofloxacin. Pyuria (P < 0.001) and glucosuria (P < 0.05) had a meaningful relationship with bacteriuria but no association was evident between age (P < 0.45), duration of diabetes (P < 0.09), macroalbuminuria (P < 0.10) and HbA_1c _level (P < 0.75), and the presence of ASB.

**Conclusion:**

The prevalence of ASB is higher in women with type 2 diabetes, for which pyuria and glucosuria can be considered as associates. Routine urine culture can be recommended for diabetic women even when there is no urinary symptom.

## Background

Diabetes leads to several abnormalities of the host defense system, and higher glucose concentration in urine may serve as a culture medium for pathogenic microorganisms as well. The risk of developing infection in diabetic patients is higher [1,2] and urinary tract is the most common site for infection [3,4]. Serious complications of urinary infection, such as emphysematous cystitis, pyelonephritis, renal or perinephric abscess, bacteremia and renal papillary necrosis occur more commonly in diabetic patients [5]. Many Urinary Tract Infections (UTIs) are asymptomatic and whether the symptomatic UTIs are preceded by asymptomatic bacteriuria (ASB) is not known [6,7]. Development of ASB in diabetic woman is much more common than in nondiabetic woman [4]. Various risk factors for ASB in women with diabetes have been suggested including sexual intercourse, age, and duration of metabolic control and complications of diabetes [7-13]. One of the biggest studies on ABS and diabetes was done by Geerlings et al in 2000 [14]. In that study, which was done on 636 nonpregnant women, the prevalence of ASB was 29% in type 2 diabetes. Risk factors for ASB in type 2 diabetic women included age, macroalbuminuria, low BMI, and UTI during the previous year. Due to the importance of UTI complications, observed to a greater extent in diabetic patients, the aim of this study was to determine the prevalence of and the associates for ASB in Iranian diabetic women. Also, this study was done to assess the organisms responsible for UTI in this group and the antimicrobial sensitivity pattern of such organisms.

## Methods

This was a cross sectional, descriptive and analytic study. Participants were recruited between February 10, 2004 and October 15, 2004 from type 2 diabetic women who had referred to Tehran Heart Center OPD (Out Patient Department) clinics. Patients were asked by their treating physicians to participate voluntarily with a response rate of 92%. Inclusion criteria considered as women with type 2 diabetes mellitus. Exclusion criteria was pregnancy, recent hospitalization or surgery (< 4 month), known urinary tract abnormalities (including cystopathy or recent urinary tract instrumentation), symptoms of UTI (including dysuria, frequency, fever, urgency, abdominal discomfort, etc) or the use of antimicrobial drugs in the last 14 days. All patients were interviewed and their medical histories were obtained using a standardized questionnaire. Also, laboratory values such as blood urea nitrogen, serum creatinin, glucose and glycosylated hemoglobin A_1c _were obtained while patients were fasting. Urine samples were checked for Macroalbuminuria by dip stick. Mid-stream clean voiding urinary specimens were collected for urinalysis, microscopy, culture and sensitivity. The specimens were refrigerated immediately and cultured within two hours. All urine samples were cultured on Blood and MacConkey agar plates. The plates were incubated at 37°C aerobically for 48 hours.

Bacteriuria was defined as the presence of at least 10^5 ^colony forming units/ml of 1 or 2 bacterial species in a culture of clean-voided midstream urine confirmed by a second culture. Presence of at least three different microorganisms in a urine specimen was considered as contamination.

According to the 1998 WHO criteria, diabetes mellitus was defined as fasting glucose concentration of at least 6.1 m mol/ l (110 mg/dl) or a two-hour post prandial glucose concentration of at least 10.0 m mol (180 mg/dl) or the use of glucose-lowering medication (tablets or insulin)[5]. Type 2 diabetes was defined as the combination of resistance to insulin action and an inadequate compensatory insulin secretory response [16].

Sample size of this study was calculated based on

N=z21−α2×pqd2
 MathType@MTEF@5@5@+=feaafiart1ev1aaatCvAUfKttLearuWrP9MDH5MBPbIqV92AaeXatLxBI9gBaebbnrfifHhDYfgasaacH8akY=wiFfYdH8Gipec8Eeeu0xXdbba9frFj0=OqFfea0dXdd9vqai=hGuQ8kuc9pgc9s8qqaq=dirpe0xb9q8qiLsFr0=vr0=vr0dc8meaabaqaciaacaGaaeqabaqabeGadaaakeaacqWGobGtcqGH9aqpdaWcaaqaaiabdQha6naaCaaaleqabaGaeGOmaidaaOGaeGymaeJaeyOeI0YaaSaaaeaaiiGacqWFXoqyaeaacqaIYaGmaaGaey41aqRaemiCaaNaemyCaehabaGaemizaq2aaWbaaSqabeaacqaIYaGmaaaaaaaa@3D6D@ p = 0.2, q = 0.8, d = 0.056, α = 0.05

which was equal to 200.

Differences between patients with and without ASB were obtained through t test for continuous variables (age, duration of diabetes, HbA_1c_, BUN and creatinine). For nominal variables we used chi squared and Fisher exact test. Mann-Whitney U test was used for dichotomous and ordinal variables (albuminuria, glucosuria and pyuria). Data were analyzed (univariate analysis) by SPSS statistical software and P value of < 0.05 was considered significant. Mean values are reported as mean ± standard deviation.

This study was approved by the Medical Ethics Committee of Tehran Heart Center and Informed Consent was obtained from each patient before the study.

## Results

Participants of this study were 202 type 2 diabetic women. Their ages ranged from 31 to 78 years with a mean of 56 ± 6.1. Duration of diabetes in this group was between 6 months to 25 years with a mean of 5.4 ± 1.2. Table 1 shows demographic data of these participants. Twenty two participants (10.9%) had two positive urine cultures with the same microorganisms. Table 2 shows isolated microorganisms. As this table shows, E. coli was the most common microorganism (59.1%) and concomitant growth of E. coli and pseudomonas auroginosa was less prevalent. Table 3 shows the sensitivity pattern of each microorganism.

**Table 1:** Participant Characteristics

No.	202
Mean age (Y)	56 ± 6.1
Duration of diabetes (Y)	4.5 ± 1.2
Duration of diabetes (Median)	4.3
Pyuria	17%
Macroalbuminuria	12.1%
Glucosuria	22.8%
Bacteriuria	10.9%
HbA_1c>8%_	43.1%
BUN (mg/dl)	39.9 ± 7.5
Creatinine (mg/dl)	0.7 ± 0.2

**Table 2:** The isolated microorganisms from urine cultures.

Microorganisms	No (%)
E. Coli	13 (59.1%)
Streptococcus hemolyticus group B	3(13.6%)
Staphylococcus coagulase negative	3(13.6%)
Pseudomonas auroginosa	2(9.1%)
E. Coli + Pseudomonas auroginosa	1(4.6%)
	22(100%)

**Table 3:** Urine isolates from diabetic women and their antibiotic sensitivity pattern

	E. Coli (14)		Staphylococcus Coagulase Negative (3)		Streptococcus Hemolyticus Group B (3)		Pseudomonas Auroginosa (3)	
	**Sensitive**	**Resistant**	**Sensitive**	**Resistant**	**Sensitive**	**Resistant**	**Sensitive**	**Resistant**

Amikacin	8	6	3	0	2	1	1	2
Gentamicin	8	6	2	1	2	1	1	2
Ciprofloxacin	5	9	2	1	1	2	1	2
Cephalotin	1	13	1	2	2	1	2	1
Ceftazidime	5	9	NT	NT	NT	NT	1	2
Ceftriaxone	0	1	NT	NT	NT	NT	1	2
Nitrofurantion	11	3	2	1	1	2	NT	NT
Nalidixic acid	4	10	NT	NT	NT	NT	NT	NT
Cotrimoxazole	4	10	2	1	2	1	NT	NT
Tobramycin	NT*	NT	NT	NT	NT	NT	1	2
Clindamycin	NT	NT	2	1	2	1	NT	NT
Erythromycin	NT	NT	1	2	2	1	NT	NT
Vancomycin	1	0	2	1	2	1	NT	NT
Ceftizoxime	NT	NT	NT	NT	NT	NT	0	3
Ampicillin	NT	NT	NT	NT	3	0	NT	NT

* NT = Not Tested

In this study, bacteriuria had a significant association with pyuria (more than five leukocytes /high power field) (p < 0.001). 45.5% of patients with bacteriuria also displayed pyuria and 86.5% of participants without bacteriuria showed no evidence of pyuria.

We observed a significant relationship between bacteriuria and glucosuria (P = 0.03). Among patients with bacteriuria, 59.1% had no glucosuria but 9.1%, 9.1% and 22.7% of them had trace, +1 and +2 glucosuria respectively.

Among participants without bacteriuria, 80.8% displayed no glucosuria. However, 4.5%, 8.5% and 6.2% had trace, +1 and +2 glucosuria respectively.

No significant association was found between bacteriuria and the age of the participants (p = 0.45). Among patients with bacteriuria, none was under 40 years old. Of patients between 40-49, 50-59 and more than 60 years, 19.2%, 11.2% and 9.2% had bacteriuria respectively.

No evidence of a significant relation between bacteriuria and the duration of diabetes (p = 0.09) was found. Among patients with less than 10 years of diabetes, 10.1% (n = 15), and patients with 10-20 years of diabetic history, 20.6% (n = 7) had bacteriuria. But among the 13 patients who had diabetes of more than 20 years duration, none had bacteriuria. No significant relationship was found (p = 0.10) in regard to the relationship between bacteriuria and macroalbuminuria. Of the 22 patients who had positive urine culture, 77.3% (n = 17) did not have macroalbuminuria. Among 24 participants with macroalbuminuria, 20.8% (n = 5) had bacteriuria as well.

In this study, no significant association was evident between bacteriuria and HbA_1c _levels (p = 0.75). 57.1 % (n = 12) of the patients with HbA_1c _levels of less than 8 had bacteriuria, and 42.9% (n = 9) of the patients whose HbA_1c _levels were 8 or more, had also developed bacteriuria.

## Discussion

In this study 22 out of 202 (10.9%) type 2 diabetic women had ASB. This is comparable with studies by Kayima et al [17] in 1996 (11.2%) and Zhanel et al [13] in 1995 (7.9%). But prevalence of ASB in diabetic women was reported 26% in Geerlings, et al's study [14] in 2000 and 26.6% in Alebiosu et al's report [18] in 2003.

As most other previous studies, E. coli was the most prevalent microorganism (59.1%) isolated from urine cultures of our participants. In a few studies, the microorganism was different. For example Kelebsiella was the most common organism in the Alebiosu study [18].

As table 3 shows, most isolated microorganisms were resistant to Cotrimoxazole. It seems that using this drug to treat bacteriuria in diabetic women should be with caution and patients should be followed-up. Two other antibiotics we are using routinely for treatment of urinary tract infections in OPD clinics are Ciprofloxacin and Nalidixic acid. Microorganisms isolated from our cultures, displayed a fairly low sensitivity to these drugs. Further studies in this regard must be accomplished and the results should be compared with the antibiograms of nondiabetic patients.

According to our statistical analysis and with regard to the calculated P values, it seems that pyuria (p < 0.001) and glucosuria (p < 0.03) have a significant relationship with bacteriuria. So, pyuria and glucosuria can be associated with ASB in diabetic women.

Even though age is a well-known risk factor for bacteriuria in women without diabetes [19] and some studies have shown age as the most important risk factor for ASB in type 2 diabetic patients [14], but age had no significant relation with ASB in our study (p = 0.45).

Some studies have shown that a longer duration of diabetes increases the risk of developing ASB [8,13], while others could not confirm this notion [7,10,20]. In our study, duration of the disease could not be considered as an associate for ASB (p = 0.09). But according to p = 0.09 and the likelihood ratio of 0.05, it can be concluded that if the sample size of this study was larger, it could be possible to find an association in this regard.

Some studies found macro albuminuria as a risk for developing ASB [14,21], however our study did not confirm macroalbuminuria as a possible associate (p = 0.10).

In confirmation of other studies [14,21], our data indicates HbA_1c _is not an associate for ASB.

Finally, we suggest a periodical urine culture be taken from diabetic patients older than 40 years to detect ASB early on (and possibly prevent its development or persistence). Further studies need to be done to assess the prevalence of asymptomatic bacteriuria in diabetic women in other Iranian population and also to assess their antimicrobial sensitivity pattern. Long-term follow up should also be implemented for diabetic patients with ASB so that the precise conclusions could be drawn regarding the course of UTI development in the future.

## Conclusion

The prevalence of ASB is higher in women with type 2 diabetes, for which pyuria and glucosuria can be considered as associates. Routine urine culture can be recommended for diabetic women even when there is no urinary symptom.

## Competing interests

The author(s) declare that they have no competing interests.

## Authors' contributions

MAB conceived of the study, contributed in analysis and interpretation of data, reviewed the manuscript and gave final approval. LS assisted in study conception & design and involved in coordinating the research and sample collection. SHA supervised data entry, carried out the analysis and drafted the manuscript. MS contributed to study design & conception, assisted with data processing and reviewed the manuscript. EK assisted in the design of the survey, assisted with data processing and contributed in interpretation of data. SF involved in planning, coordinating the research and helped in drafting the article. All the authors read and approved the final manuscript.

## Pre-publication history

The pre-publication history for this paper can be accessed here:


